# Front-Line ICI-Based Combination Therapy Post-TKI Resistance May Improve Survival in NSCLC Patients With EGFR Mutation

**DOI:** 10.3389/fonc.2021.739090

**Published:** 2021-11-23

**Authors:** Tian Tian, Min Yu, Juan Li, Maoqiong Jiang, Daiyuan Ma, Shubin Tang, Zhiyu Lin, Lin Chen, Youling Gong, Jiang Zhu, Qiang Zhou, Meijuan Huang, You Lu

**Affiliations:** ^1^ Department of Thoracic Oncology, Cancer Center, West China Hospital, Sichuan University, Chengdu, China; ^2^ Department of Thoracic Cancer, Medical Oncology Center, Sichuan Cancer Hospital & Institute, Sichuan Cancer Center, School of Medicine, University of Electronic Science and Technology of China, Chengdu, China; ^3^ Department of Thoracic Oncology, The Second People’s Hospital of Yibin, Yibin, China; ^4^ Department of Oncology, Cancer Center, Affiliated Hospital of North Sichuan Medical College, Nan Chong, China; ^5^ Department of Oncology, The First People’s Hospital of Neijiang, Neijiang, China; ^6^ Department of Oncology and Hematology, Leshan People’s Hospital, Leshan, China; ^7^ Cancer Center, Suining Central Hospital, Suining, China

**Keywords:** non–small cell lung cancer, immune checkpoint inhibitor, tyrosine kinase inhibitor, epidermal growth factor receptor, resistance

## Abstract

**Background:**

Data on the use of immune checkpoint inhibitors (ICIs) in advanced non-small cell lung cancer (NSCLC) patients with epidermal growth factor receptor (EGFR) mutation are limited. The current study aimed to assess the efficacy of ICIs in EGFR-mutant advanced NSCLC and explore the relevant influential factors.

**Materials and Methods:**

Relevant clinical data of EGFR-mutant NSCLC patients who had received ICIs were collected from multiple hospitals. The primary endpoint was progression-free survival (PFS), and the secondary endpoints were overall survival (OS), objective response rate (ORR), and relevant influential factors.

**Results:**

A total of 122 advanced EGFR-mutant NSCLC patients were included in the final analysis. The total cohort had an objective response rate (ORR) of 32.0%, a median progression-free survival (mPFS) of 5.0 months, and a median overall survival (mOS) of 14.4 months. Among 96 patients with common EGFR mutations (19Del, 52 patients; L858R, 44 patients), those who were administered front-line ICI exhibited better survival benefits than those who received later-line ICI after disease progression on tyrosine kinase inhibitors (TKIs) treatment (mPFS: 7.2 months *vs.* 3.4 months, respectively, P < 0.0001; mOS: 15.1 months *vs.* 8.4 months, respectively, P <0.0001). Moreover, the efficacy of ICI-based combination therapy was better than that of ICI monotherapy (mPFS: 5.0 months *vs.* 2.2 months, respectively, P = 0.002; mOS: 14.4 months *vs.* 7.0 months, respectively, P = 0.001). Multivariate analysis showed that ICI-based combination therapy and front-line ICI administration after progression on EGFR-TKI were associated with significant improvements in both PFS and OS (P < 0.05). A high PD-L1 expression (tumor proportion score, TPS≥50%) and the EGFR L858R mutation were only significantly associated with a better PFS (P <0.05). A better Eastern Cooperative Oncology Group (ECOG) status was independently associated with a favorable OS (P <0.05).

**Conclusions:**

Taken together, combination immunotherapy in front-line was associated with improvement of survival in EGFR-mutant NSCLC patients post-TKI resistance. Further prospective studies with large sample sizes are required to identify the optimal combinatorial treatment strategy.

## Introduction

Epidermal growth factor receptor tyrosine kinase inhibitors (EGFR-TKIs) as a standard first-line treatment for advanced non-small-cell lung cancer harboring EGFR mutation yield great efficacy but acquired resistance and disease progression are inevitable (1–3). Salvage treatment options following available TKI failure are limited; chemotherapy serves as the primary modality with unsatisfactory efficacy (4–7). Immune checkpoint inhibitors (ICIs), such as anti-programmed cell death 1 (PD-1) and programmed cell death-ligand 1 (PD-L1) agents, have considerably improved the survival of driver gene wild-type advanced NSCLC (8–10). Although a few reports have been published recently, the role of ICI in EGFR-mutant NSCLC after EGFR-TKI failure is still controversial. Disappointing results have been demonstrated with ICI monotherapy in IMMUNOTARGET (11) and other studies (12–14), while some physicians advocate that ICI-based combination therapy may be an option (15). Subgroup analysis in the IMpower 150 study showed that the combination of paclitaxel, carboplatin, bevacizumab, and atezolizumab improved PFS but not significant OS benefit as compared to that with bevacizumab plus chemotherapy. This four-drug regimen owned an incidence of grade 3 to 4 treatment-related adverse events of 57% (16). Two studies with a combination approach have reported promising results on response rate (RR) and survival (17, 18), while flaws exist due to the small sample size and insufficient information on patients with T790M. Another study with ICI combination treatment got a worse outcome with an objective response rate (ORR) of 18.6% and a median progression-free survival (mPFS) of 2.8 months (19). Moreover, a few studies with small samples have retrospectively analyzed the data of EGFR 20 insertion mutation (EGFR 20Ins) to evaluate the efficacy of ICI (20–22). Therefore, more studies are urgent to explore the role of ICI in EGFR-mutant NSCLC patients.

This retrospective study aimed to summarize the efficacy of ICI in EGFR-mutant NSCLC after progression on TKI treatment and explore issues, such as the administration timing of ICI, whether ICI monotherapy or ICI-based combination therapy is better, and the efficacy of ICI for EGFR 20Ins.

## Materials and Methods

### Patients’ Clinical Data

The clinical data of eligible patients were extracted from the electronic medical records of seven different institutions in China (including West China Hospital of Sichuan University, Sichuan Cancer Hospital & Institute, The Second People’s Hospital of Yibin, Affiliated Hospital of North Sichuan Medical College, The First People’s Hospital of Neijiang, Leshan people’s Hospital, Suining Central Hospital) from September 2016 to May 2020. The inclusion criteria were as follows: treatment with ICI (anti-PD-1/PD-L1 inhibitor); a pathological diagnosis of NSCLC and at stage IV according to tumor size, lymph node, and metastasis (TNM) stages; exhibition the activation of EGFR mutations on exons 18 to 21. Patients who had participated in clinical trials or had other cancers were excluded. Related baseline demographic variables, including sex, age, Eastern Cooperative Oncology Group (ECOG) performance status, immunotherapy strategy, smoking history, sites of metastasis, histological type, and prior treatment information, were collected. This study adhered to the tenets of the Declaration of Helsinki and was performed following the principles of good clinical practice and approved by the institutional ethical review board. As only anonymous medical records of patients were used, the requirement for informed consent was waived by the ethical committee.

### EGFR Mutation and PD-L1 Analysis

Tumor tissue samples obtained from biopsy, resection, and cytology were used for immunohistochemical detection. PD-L1 status was determined by immunohistochemistry analyses (23), and EGFR mutations were evaluated by polymerase chain reaction or next-generation sequencing (24), which was used according to standard protocols of the respective centers. The PD-L1 tumor proportion score (TPS) refers to the percentage of tumor cells showing partial or complete membrane staining (25). PD-L1 expression≥50% was classified as a strong positive result (26). All gene alterations and PD-L1 expression status were part of the patients’ clinical information at baseline.

### Statistical Analysis

Categorical variables are presented as numbers and percentiles, whereas continuous variables are presented as medians and ranges. Each patient’s response to ICI treatment was assessed using the Response Evaluation Criteria in Solid Tumors (RECIST) v.1.1. PFS was defined as the time from treatment initiation to disease progression or death from any cause. The patients still alive at the date of last follow-up visit (April 1, 2021) were censored. Kaplan–Meier survival curves were constructed for PFS and OS, and the differences between groups were identified using the log-rank test. The Cox proportional hazards regression model was used for univariate and multivariate analyses. The follow–up time was calculated using the reverse Kaplan–Meier method. Two-tailed P values were calculated for all analyses and statistical significance was set at P<0.05. All statistical analyses were performed using SPSS 25.0 and GraphPad 8.0 statistical software.

## Results

### Patient Characteristics

A total of 122 eligible patients with EGFR-mutant NSCLC were finally included. The median follow-up time was 15.4 months (range: 0.6–28.8 months) and median age was 56 years (range: 30–85 years). The majority of the patients had a good performance status (ECOG = 0–1; 105/122, 86.1%) and were diagnosed with adenocarcinoma (112/122, 91.8%). EGFR mutation subtypes consisted of EGFR exon 19 deletion (19Del) (N = 52, 42.6%), EGFR exon 21 L858R mutation (EGFR 21 L858R) (N = 44, 36.1%), EGFR 20Ins (N = 23, 18.9%), and three other patients had uncommon mutations (G719X, N = 2; L861Q, N = 1). 69 of patients carrying EGFR common mutation (19Del, 21 L858R) underwent gene re-test after first or second-generation TKI treatment, with 31 cases acquired T790M mutation. 43 cases with common EGFR mutation were treated with osimertinib after progression on first and second-generation TKI. Most patients received an anti-PD-1 agent (116/122, 95.1%). The PD-L1 expression status was known in 86 patients (86/122, 70.5%). Further details of patients’ characteristics are shown in **Table 1**.

**Table 1:** Characteristics of NSCLC patients with EGFR mutation treated with the immune checkpoint inhibitors.

Clinical Characteristic	All EGFR (*N* = 122)	EGFR^19Del^ (*N* = 52)	EGFR^L858R^ (*N* = 44)	EGFR^20Ins^ (*N* = 23)	EGFR ^Other*^ (*N* = 3)
**Age, median (range)**	56 (30~85)	55 (39~71)	56.5 (30~82)	58 (35~85)	49 (43~70)
**Age**					
>65	24 (19.7%)	11 (21.2%)	9 (20.5%)	3 (13.0%)	1 (33.3%)
≦65	98 (80.3%)	41 (78.8%)	35 (79.5%)	20 (87.0%)	2 (66.7%)
**ECOG**					
0	44 (36.1%)	15 (29.0%)	18 (40.9%)	9 (39.1%)	2 (66.7%)
1	61 (50.0%)	28 (54.0%)	20 (45.5%)	12 (52.2%)	1 (33.3%)
≧2	17 (13.9%)	9 (17.0%)	6 (13.6%)	2 (8.7%)	0 (0.0%)
**Gender**					
Male	59 (48.4%)	29 (55.8%)	15 (34.1%)	12 (52.2%)	3 (100%)
Female	63 (51.6%)	23 (44.2%)	29 (65.9%)	11 (47.8%)	0 (0.0%)
**Smoking**					
Current/Former	33 (27.0%)	16 (30.8%)	8 (18.2%)	7 (30.4%)	2 (66.7%)
Never	89 (73.0%)	36 (69.2%)	36 (81.8%)	16 (69.6%)	1 (33.3%)
**Histology**					
Adenocarcinoma	112 (91.8%)	48 (92.3%)	42 (95.5%)	19 (82.6%)	3 (100%)
Squamous cell carcinoma	10 (8.2%)	4 (7.7%)	2 (4.5%)	4 (17.4%)	0 (0.0%)
**Metastasis Site**					
brain	49 (40.2%)	22 (42.3%)	20 (45.5%)	7 (30.4%)	0 (0.0%)
bone	61 (50.0%)	26 (50.0%)	21 (47.7%)	13 (56.5%)	1 (33.3%)
liver	23 (18.9%)	11 (21.2%)	6 (13.6%)	6 (26.1%)	0 (0.0%)
**The line of ICI**					
1	9 (7.4%)	0 (0.0%)	0 (0.0%)	9 (39.1%)	0 (0.0%)
2	31 (25.4%)	11 (21.2%)	12 (27.3%)	6 (26.1%)	2 (66.7%)
3	42 (34.4%)	21 (40.4%)	15 (34.1%)	5 (21.7%)	1 (33.3%)
≧4	40 (32.8%)	20 (38.4%)	17 (38.6%)	3 (13.0%)	0 (0.0%)
**ICI Treatment**					
ICI Monotherapy	32 (26.2%)	12 (23.1%)	12 (27.3%)	8 (34.8%)	0 (0.0%)
ICI-based combination therapy	90 (73.8%)	40 (76.9%)	32 (72.7%)	15 (65.2%)	3 (100%)
**ICI Drug**					
PD-1	116 (95.1%)	50 (96%)	40 (90.9%)	23 (100%)	3 (100%)
PD-L1	6 (4.9%)	2 (4.0%)	4 (9.1%)	0 (0.0%)	0 (0.0%)
**PD-L1 expression**					
<1%	24 (19.7%)	12 (23.1%)	6 (13.6%)	5 (21.7%)	1 (33.3%)
1-49%	28 (23.0%)	14 (26.9%)	6 (13.6%)	8 (34.8%)	0 (0.0%)
≧50%	34 (27.8%)	14 (26.9%)	17 (38.6%)	2 (8.7%)	1 (33.3%)
unknown	36 (29.5%)	12 (23.1%)	15 (34.1%)	8 (34.8%)	1 (33.3%)

*G719X 2, L861Q 1.

### Survival of EGFR-Mutant Patients

**Figure 1:**
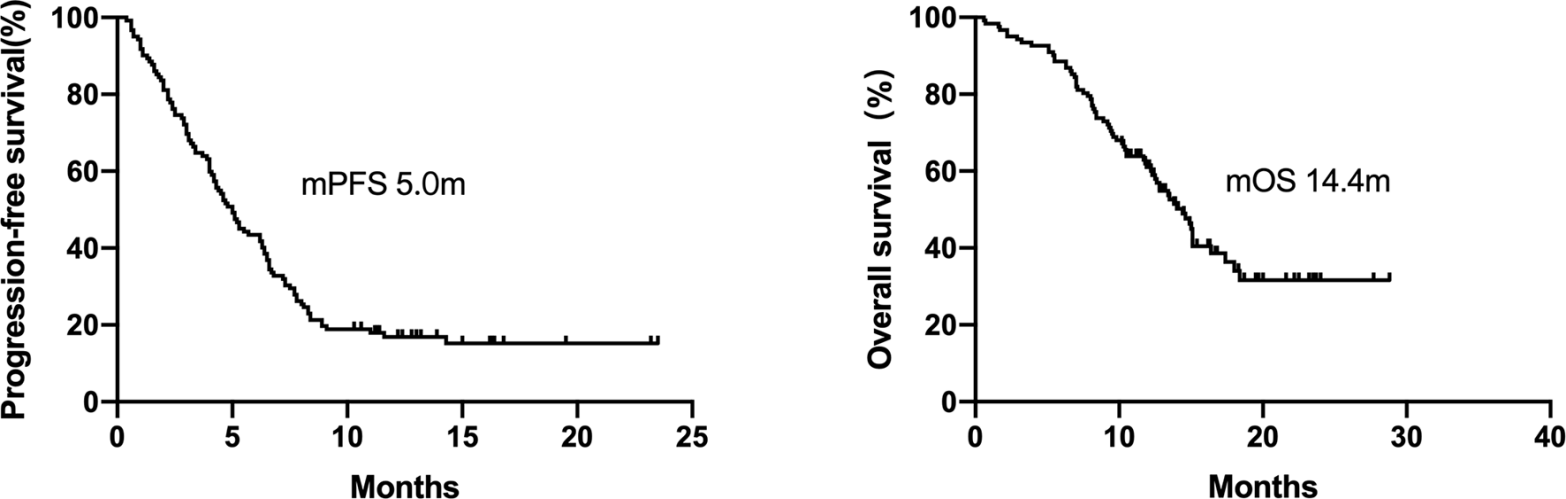
Kaplan–Meier curves for progression-free survival and overall survival of patients with EGFR-mutant NSCLC (n = 122). The median PFS and OS were 5.0 months and 14.4 months, respectively.

The ORR of the total 122 patients was 32.0% (39/122), and the disease control rate was 70.0% (85/122). The median PFS (mPFS) and OS (mOS) were 5.0 months (95% CI = 4.1–5.8 months) and 14.4 months (95% CI = 12.5–16.4 months), respectively (**Figure 1** and **Table 2**).

**Table 2:** The treatment responses of different EGFR mutation types.

RECISTResponse	All patients (n = 122)	Common mutations (n = 96)	19Del (n = 52)	L858R (n = 44)	Uncommon mutations (n = 26)	20Ins (n = 23)	Other* (n = 3)
Complete response	0	0	0	0	0	0	0
Partial response	39 (32%)	30 (31.3%)	16 (30.8%)	14 (31.8%)	9 (34.6%)	8 (34.8%)	1 (33.3%)
Stable disease	46 (37.7%)	37 (38.5%)	16 (30.8%)	21 (47.7%)	9 (34.6%)	8 (34.8%)	1 (33.3%)
Progressive disease	37 (30.3%)	29 (30.2%)	20 (38.5%)	9 (20.5%)	8 (30.8%)	7 (30.4%)	1 (33.3%)
Overall response rate	32.0%	31.3%	30.8%	31.8%	34.6%	34.8%	33.3%
Disease control rate	70.0%	70.0%	61.5%	79.5%	69.2%	69.6%	66.7%
Median progression-free survival, months	5.0	4.4	3.8	6.1	6.4	6.4	7.7
Median overall survival, months	14.4	13.4	12.8	13.5	NR	NR	18.4

*G719X 2, L861Q 1.

NR, not reached.

The group with common EGFR mutations (19Del and L858R) had an ORR, mPFS and mOS of 31.3% (30/96), 4.4 months (95%CI = 3.7–5.1 months) and 13.4 months (95%CI = 11.7–15.1 months), respectively (**Table 2**).

ICI for patients carrying EGFR 20Ins displayed an ORR of 34.8% (8/23) and a median PFS of 6.4 months (95%CI = 4.8–8 months); the median OS was not reached. Among another three patients with uncommon EGFR mutations [L861Q (1patient), G719X (2 patients)], the median PFS and OS were 7.7 months and 18.4months, respectively (**Table 2**).

### Clinical Features Associated With Outcomes in Patients With Common EGFR Mutations

All 96 patients with common EGFR mutations (19Del and L858R) had previously been treated with EGFR-TKIs. All patients with prior TKI treatment failure who carried the acquired T790M mutation have received osimertinib. Further analyses of clinical features were subsequently performed to identify the benefitting population.

**Figure 2:**
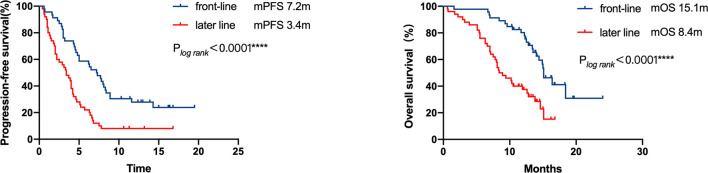
Kaplan–Meier curves of progression-free survival and overall survival of patients who received ICI therapy at different lines of treatment. The patients who were administered front-line ICI exhibited superior survival benefits than those who received ICI as later line after progression on TKI (mPFS 7.2 months *vs.* 3.4 months, respectively, P < 0.0001; mOS 15.1 months *vs.* 8.4 months, respectively, P < 0.0001).

46 patients were immediately administered ICI after progression on TKI, which was defined as front-line ICI post-TKI progression, whereas the remaining 50 patients received later-line ICI because they received other systemic therapy regimens in the interval between TKIs and ICI treatment. The patients who received front-line ICI showed enhanced survival benefits compared to those who received ICI as a later line post-TKIs progression (mPFS, 7.2 months [95% CI = 5.4–9 months], *vs.* 3.4 months [95% CI = 2.2– 4.5 months], respectively, P <0.0001; mOS, 15.1 months [95% CI = 13.5–16.7 months], *vs.* 8.4m [95% CI = 6.2–10.6 months], respectively, P<0.0001; **Figure 2**). The group treated with front-line ICI had a better ECOG performance score and higher PD-L1 expression than the group treated with later-line ICI.

**Figure 3:**
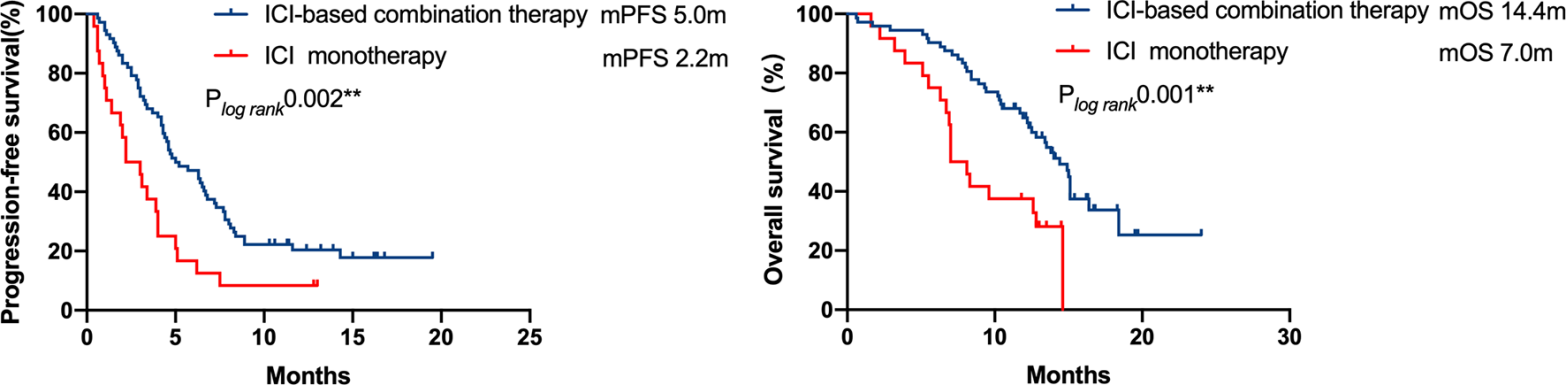
Kaplan–Meier curves of progression-free survival and overall survival of patients who received ICI-based combination therapy *versus* ICI monotherapy. The efficacy of ICI-based combination therapy was better than that of ICI monotherapy (mPFS 5.0months *vs.* 2.2 months, P = 0.002, mOS 14.4 months *vs.* 7.0 months, P = 0.001).

A total of 72 patients were treated with ICI-based combination therapy: 50 received a combination of ICI with chemotherapy, 8 received a combination of ICI with chemotherapy and radiotherapy, 12 received a combination of ICI with chemotherapy plus an anti-angiogenic agent, and 2 received dual ICIs (an anti-PD-1 agent combined with an anti-cytotoxic-T-lymphocyte-associated protein 4 inhibitor). The efficacy of ICI-based combination therapy was better than that of ICI monotherapy (mPFS, 5.0 months [95% CI 3.2–6.8 months) *vs.* 2.2 months [95% CI = 0.9–3.5 months], respectively, P = 0.002; mOS, 14.4 months [95% CI = 12.8–16 months] *vs.* 7.0 months [95% CI = 5.6–8.3 months], respectively, P=0.001; **Figure 3**).

**Figure 4:**
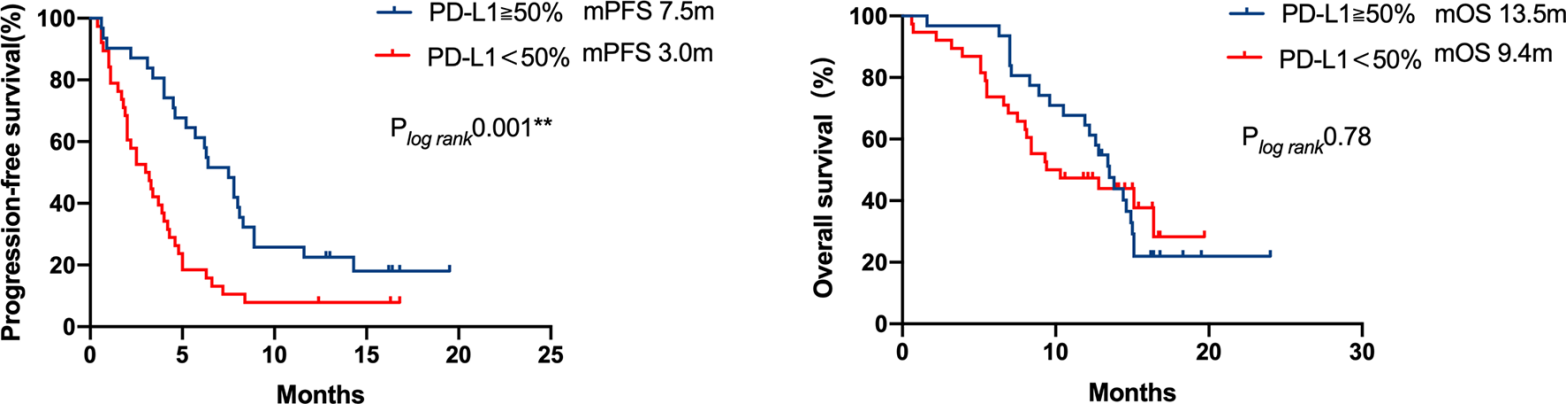
Kaplan–Meier curves of progression-free survival and overall survival of patients with differential PD-L1 expression. A significant PFS benefit was observed in patients with strong positive PD-L1 expression (TPS ≥ 50%) compared with that in patients with a lower PD-L1 expression (TPS < 50%) (7.5 months *vs* 3.0 months, respectively, P = 0.001), but the difference in OS was not statistically significant.

In patients with available PD-L1 expression data (n = 69/96, 71.9%), 31 patients exhibited strongly positive PD-L1 expression (TPS≥50%), whereas 38 patients presented PD-L1 expression less than 50%. A significant PFS benefit was observed in patients with strongly positive PD-L1 expressions (TPS≥50%) compared with the cohort with a lower PD-L1 expression (TPS<50%) (7.5 *vs.* 3.0 months, respectively, P = 0.001). However, the difference in OS was not statistically significant (**Figure 4**).

**Figure 5:**
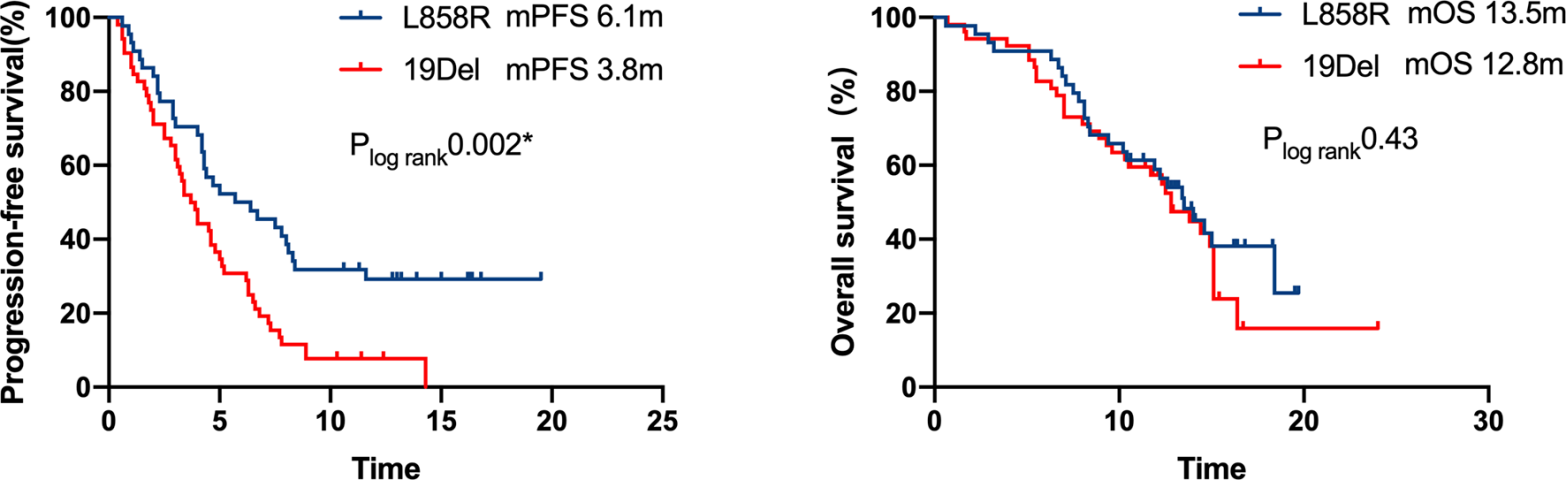
Kaplan–Meier curves of progression-free survival and overall survival of patients with differential mutation type. A significant PFS benefit was observed in patients with L858R compared with that in patients with 19Del (6.1 months *vs.* 3.8 months, respectively, P = 0.002), but the difference in OS was not statistically significant.

A multivariate analysis was performed by including factors that were found to be significant in the univariate analysis (P<0.05) and those considered to be clinically significant (**Table 3**). The results indicated that strongly positive PD-L1 expression (TPS≥50%), ICI-based combination therapy, front-line ICI treatment after EGFR TKI progression, and the EGFR L858R genotype were all significantly associated with improved PFS (P <0.05) (**Figure 5**). A good ECOG status, ICI-based combination therapy, and front-line ICI treatment after EGFR TKI progression were found to be independently associated with a favorable OS, after adjusting for other clinical factors (P <0.05; **Table 3**).

**Table 3:** The univariable and multivariable analyses of PFS and OS among the EGFR common mutation population.

	Univariable analysis				Multivariable analysis			
	PFS		OS		PFS		OS	
	HR (95%CI)	*P*	HR (95%CI)	*P*	HR (95%CI)	*P*	HR (95%CI)	*P*
**Age** ≦65 *vs* >65	1.01 (0.58~1.78)	0.969	0.89 (0.66~1.22)	0.47	1.08 (0.56~2.083)	0.819	0.57 (0.27~1.17)	0.123
**Smoking status** Yes *vs* No	1.22 (0.74~2.01)	0.437	1.02 (0.56~1.86)	0.953	0.93 (0.56~1.57)	0.792	1.03 (0.55~1.93)	0.916
**ECOG score** 0>1 *vs* ≧2	0.74 (0.56~1.00)	0.048	0.54 (0.39~0.75)	<0.0001	0.62 (0.32~1.21)	0.161	0.33 (0.16~0.71)	0.004
**PD-L1 expression** ≧50% *vs *<50%	0.42 (0.25~0.71)	0.001	0.92 (0.52~1.65)	0.782	0.49 (0.28~0.88)	0.017	1.45 (0.75~2.78)	0.271
**Treatment strategy** Combined *vs* Mono	0.46 (0.28~0.75)	0.002	0.39 (0.22~0.70)	0.002	0.38 (0.21~0.68)	0.001	0.47 (0.25~0.91)	0.024
**Time of ICI treatment** front-line *vs* later line	0.64 (0.51~0.80)	<0.0001	0.58 (0.44~0.77)	<0.0001	0.53 (0.31~0.92)	0.024	0.35 (0.17~0.69)	0.003
**EGFR mutation subtype** L858R *vs* 19Del	0.49 (0.31~0.77)	0.002	0.81 (0.48~1.36)	0.422	0.49 (0.30~0.79)	0.004	0.70 (0.40~1.23)	0.219

## Discussion

Chemotherapy, as the most common subsequent treatment regimen after the discontinuation of EGFR-TKI therapy, has limited benefits for EGFR-mutant NSCLC patients (4–7). A retrospective study indicated that 27% of patients received chemotherapy combined with ICI after the failure of osimertinib (27), suggesting that the efficacy of salvage chemotherapy alone was unsatisfactory, and thus physicians were enthusiastic to explore ICI in EGFR-mutant NSCLC. Although the role of ICI monotherapy uses in EGFR-mutant NSCLC is debatable (11–14), our study indicated that ICI treatment for EGFR-mutant NSCLC obtained a mPFS of 5 months and a mOS of 14.4 months. In detail, ICI-based combination therapy outperformed ICI monotherapy, with a mPFS of 5 months versus 2.2 months and mOS of 14.4 months versus 7 months, respectively. These results were somewhat interesting.

Previous single-arm studies on ICI-based combination regimens in EGFR-mutant NSCLC patients after EGFR TKI failure have reported inconsistent results (17–19). The CT 18 study (18) and other studies using a combination approach of ICI with chemotherapy have exhibited survival benefits (16, 17), which was also observed in our study, whereas a study with camrelizumab plus apatinib achieved inferior outcome (19). Basic studies support that chemotherapy, antiangiogenic drugs, and radiotherapy exert synergistic effects with ICI *via* positive regulation of the immune system, changing the tumor immune microenvironment, and releasing tumor neoantigens (28–32). Besides the role of ICI, the optimal combination strategy is still unclear. Our study including patients who received first–, second–, third-generation EGFR TKI in the first-line or after acquired T790M mutation reflected the real-world situation, and the majority of cases received ICI combined with chemotherapy. The current study evaluated the efficacy of ICI combination regimen versus monotherapy and observed improved survival from ICI-based combination therapy. Considering the toxicities of ICI combined with chemotherapy (16), an alternative combined partner from chemotherapy to antiangiogenic drugs seems reasonable (19), but the efficacy of the chemotherapy-free combined strategy needed to be further explored.

Several studies concerning gene wild-type NSCLC have indicated that the earlier the use of ICIs, the better the outcome maybe (33, 34). Some studies on EGFR-mutant NSCLC after EGFR TKI failure recruited patients without prior palliative chemotherapy (17, 18), whereas a study of camrelizumab plus apatinib (19) including patients in the later setting exhibited lower RR and shorter PFS. Our study showed that front-line administration of ICI after EGFR-TKI resistance was more beneficial in terms of PFS and OS. This phenomenon is consistent with that observed in patients with wild-type driver gene. Although the tumor microenvironment (TME) of EGFR-mutant NSCLC is immunosuppressive (28), EGFR-TKI may activate the TME by increasing dendritic cells and CD8+ cells, reducing Tregs, and inhibiting M2-like macrophages polarization at an early stage (35). EGFR-TKI could also affect the expression of PD-L1 (36) and the distribution of the CD4+, and Foxp3+ cells within the TME (37–39). We speculate that the insertion of other treatments before ICI may possibly perturb the favorable immune microenvironment that may exist after TKI treatment. Therefore, the administration timing of ICI treatment for this population may be also important.

The predictive effect of PD-L1 status on the efficacy of ICI treatment among EGFR-mutant NSCLC patients is inadequate and debatable. It is thought that the PD-L1 expression in EGFR-mutant NSCLC patients is mainly mediated by carcinogenic signaling pathways rather than an adaptive immune process, resulting in a lowered capacity to predict the efficacy of immunotherapy (40, 41). Some studies have found that the status of PD-L1 expression could not be used to screen out ICI responders in EGFR-mutant NSCLC patients (42). On the contrary, other studies demonstrated that ICIs can also be used for EGFR-mutant NSCLC patients who have high PD-L1 expression (43). In cohort 1 (n=111) of the ALTLANTIC study (44), durvalumab was used as the third or later line treatment for advanced EGFR/ALK-positive NSCLC patients. Patients with PD-L1 expression ≥ 25% had an ORR of 12%, and better median PFS and 2-year OS rates than patients with PD-L1 expression < 25% (13.3 months *vs.* 9.9 months, 40.7% *vs.* 14.7%, respectively). Similarly, the results of our study indicated that PD-L1 expression ≥50% at baseline was related to better PFS of ICI treatment. EGFR L858R was found to be associated with favorable PFS in our study, which is consistent with the finding of a previous study (45).

It must be noted that this study has certain limitations. First, the results should be interpreted with caution because of the retrospective nature of the study. Second, PD-L1 expression data were not available for every individual. Finally, we could not obtain the PD-L1 expression status data after EGFR-TKI discontinuation, which may be more accurate to predict the efficacy of ICI treatment. Despite these limitations, this retrospective study was performed rigorously and ethically to provide a certain reference value for clinical practice.

In conclusion, ICI therapy, especially front-line ICI therapy and ICI-based combination therapy, may be beneficial for improving the prognosis of advanced EGFR-mutant NSCLC patients after EGFR-TKI therapy discontinuation. These findings need to be verified by prospective randomized controlled phase III clinical studies.

## Data Availability Statement

The raw data supporting the conclusions of this article will be made available by the authors, without undue reservation.

## Ethics Statement

The studies involving human participants were reviewed and approved by the institutional ethical review board. Written informed consent for participation was not required for this study in accordance with the national legislation and the institutional requirements.

## Author Contributions

Study design and data analysis: TT, MY, and MH. Data collection: TT, MY, MH, JL, MJ, DM, ST, ZL, LC, YG, JZ, QZ, and YL. Paper writing: TT. Manuscript modification: MY, and MH. All authors contributed to the article and approved the submitted version.

## Conflict of Interest

The authors declare that the research was conducted in the absence of any commercial or financial relationships that could be construed as a potential conflict of interest.

## Publisher’s Note

All claims expressed in this article are solely those of the authors and do not necessarily represent those of their affiliated organizations, or those of the publisher, the editors and the reviewers. Any product that may be evaluated in this article, or claim that may be made by its manufacturer, is not guaranteed or endorsed by the publisher.

## Abbreviations

EGFR, epidermal growth factor receptor; TKI, tyrosine kinase inhibitors; ICI, immune checkpoint inhibitors; NSCLC, non-small-cell lung cancer; TMN stages, tumor size, lymph node, and metastasis; PFS, progression-free survival; OS, overall survival; ORR, objective response rate; DCR, disease control rate; EGFR 20Ins, EGFR Exon 20 insertion; EGFR19 Del, EGFR exon 19 deletion; EGFR 21 L858R, EGFR exon 21 L858R mutation; T790M, T790M mutation.
